# A point mutation in the ion conduction pore of AMPA receptor *GRIA3* causes dramatically perturbed sleep patterns as well as intellectual disability

**DOI:** 10.1093/hmg/ddx270

**Published:** 2017-07-14

**Authors:** Benjamin Davies, Laurence A Brown, Ondrej Cais, Jake Watson, Amber J Clayton, Veronica T Chang, Daniel Biggs, Christopher Preece, Polinka Hernandez-Pliego, Jon Krohn, Amarjit Bhomra, Stephen R F Twigg, Andrew Rimmer, Alexander Kanapin, The WGS500 Consortium, Arjune Sen, Zenobia Zaiwalla, Gil McVean, Russell Foster, Peter Donnelly, Jenny C Taylor, Edward Blair, David Nutt, A Radu Aricescu, Ingo H Greger, Stuart N Peirson, Jonathan Flint, Hilary C Martin

**Affiliations:** 1 Wellcome Trust Centre for Human Genetics, University of Oxford, Oxford, Oxfordshire OX3 7BN, UK; 2 Nuffield Department of Clinical Neurosciences, Sleep and Circadian Neuroscience Institute, University of Oxford, Oxford, Oxfordshire OX3 9DU, UK; 3 Medical Research Council (MRC) Laboratory of Molecular Biology, Neurobiology Division, Cambridge, Cambridgeshire CB2 0QH, UK; 4 Clinical Genetics Group, Weatherall Institute of Molecular Medicine, University of Oxford, Oxford, Oxfordshire OX3 9DS, UK; 5 Institute for Cancer Research, London SM2 5NG, UK; 6 Department of Oncology, University of Oxford, Oxford, Oxfordshire OX3 7DQ, UK; 7 Oxford Epilepsy Research Group, NIHR Biomedical Research Centre, Nuffield Department of Clinical Neuroscience, John Radcliffe Hospital, Oxford OX3 9DU, UK; 8 Department of Neuroscience, John Radcliffe Hospital, Oxford, Oxfordshire OX3 9DU, UK; 9 Big Data Institute, Li Ka Shing Centre for Health Information and Discovery, University of Oxford, Oxford, Oxfordshire OX3 7FZ, UK; 10 Department of Statistics, University of Oxford, Oxford, Oxfordshire OX1 3LB, UK; 11 National Institute for Health Research Oxford Biomedical Research Centre (NIHR Oxford BRC), Oxford, Oxfordshire OX3 7LE, UK; 12 Department of Clinical Genetics, Oxford University Hospitals NHS Trust, Oxford, Oxfordshire OX3 7HE, UK; 13 Division of Brain Sciences, Department of Medicine, Centre for Neuropsychopharmacology, Imperial College London, London W12 0NN, UK; 14 Center for Neurobehavioral Genetics, Semel Institute for Neuroscience and Human Behavior, University of California-Los Angeles, CA 90095, USA; 15 Wellcome Trust Sanger Institute, Hinxton, Cambridgeshire CB10 1SA, UK

## Abstract

The discovery of genetic variants influencing sleep patterns can shed light on the physiological processes underlying sleep. As part of a large clinical sequencing project, WGS500, we sequenced a family in which the two male children had severe developmental delay and a dramatically disturbed sleep-wake cycle, with very long wake and sleep durations, reaching up to 106-h awake and 48-h asleep. The most likely causal variant identified was a novel missense variant in the X-linked *GRIA3* gene, which has been implicated in intellectual disability. *GRIA3* encodes GluA3, a subunit of AMPA-type ionotropic glutamate receptors (AMPARs). The mutation (A653T) falls within the highly conserved transmembrane domain of the ion channel gate, immediately adjacent to the analogous residue in the *Grid2* (glutamate receptor) gene, which is mutated in the mouse neurobehavioral mutant, *Lurcher*. *In vitro*, the *GRIA3*(A653T) mutation stabilizes the channel in a closed conformation, in contrast to *Lurcher*. We introduced the orthologous mutation into a mouse strain by CRISPR-Cas9 mutagenesis and found that hemizygous mutants displayed significant differences in the structure of their activity and sleep compared to wild-type littermates. Typically, mice are polyphasic, exhibiting multiple sleep bouts of sleep several minutes long within a 24-h period. The *Gria3*^A653T^ mouse showed significantly fewer brief bouts of activity and sleep than the wild-types. Furthermore, *Gria3*^A653T^ mice showed enhanced period lengthening under constant light compared to wild-type mice, suggesting an increased sensitivity to light. Our results suggest a role for GluA3 channel activity in the regulation of sleep behavior in both mice and humans.

## Introduction

Sleep is a complex physiological process involving the coordinated interaction of multiple neurotransmitter systems and a diverse network of arousal and sleep-promoting neurons (1). Sleep is characterized at a behavioral level by changes in rest/activity, body posture and responsiveness to stimuli (2) and at a neurophysiological level by vigilance states defined by the electroencephalogram (EEG), including waking, rapid eye movement (REM) and non-rapid eye movement (NREM) sleep. The timing and structure of sleep/wake behavior is determined by two interacting processes (3). The homeostatic process increases during periods of wakefulness, and an individual deprived of sleep will show a compensatory increase in subsequent amount of sleep. In turn, a circadian drive for arousal opposes the increasing propensity to sleep across the day (4). In mammals, the circadian process involves the expression of a number of key clock genes in the master circadian pacemaker located in the hypothalamic suprachiasmatic nuclei (SCN) (5). By contrast, the homeostatic process is poorly understood.

Over the last 25 years, progress has been made in dissecting the genetic mechanisms affecting the circadian clock and sleep homeostasis. Early quantitative trail loci (QTL) mapping studies in mice revealed several candidate loci and indicated a polygenic basis for the amount of sleep and its organization (6). Similar QTL studies have also identified genetic determinants of circadian period (7). Additionally, forward-genetic screens have been critical in the identification of the molecular clock genes in mice and flies (8–10), and more recently, they have been used to identify two genes involved in regulating sleep and wakefulness in mice (11) (encoding the SIK3 protein kinase and the sodium leak channel NALCN).

Several genes have been found to cause rare circadian rhythm and sleep disorders in humans (12). For example, Advanced Sleep Phase Syndrome arises from mutations in the molecular clock genes Period 2 (*PER2*) (13), Period 3 (*PER3*) (14), and *CSNK1D* (15). Disruption of the orthologs of these genes in mice also alters their circadian rhythm (14–16). Recently, twelve new loci affecting chronotype (“morningness” or “eveningness” preference) were identified through a genome-wide association study in the UK Biobank (17), including four in or near genes known to be involved in circadian rhythms (*PER2, APH1A, RGS16*, and *FBXL13*).

Glutamate, a major excitatory neurotransmitter, is known to play a key role in the regulation of sleep. In rats, wakefulness and sleep are characterized by changes in extracellular glutamate levels (18,19), as well as changes in the level and phosphorylation state of GluA1-containing alpha-amino-3-hydroxy-5-methylisoxazole-4-propionate (AMPA)-type ionotropic glutamate receptors (AMPARs) (encoded by *Gria1*) (20), important mediators of synaptic transmission and plasticity (21). Furthermore, glutamate is the principal neurotransmitter of the retino-hypothalamic tract which conveys light information from the eye to the SCN clock (22).

Here we report a pathogenic mutation in the *GRIA3* gene, encoding the GluA3 subunit of AMPARs, found in a family with an apparently novel disorder: severe sleep and circadian rhythm disruption, with a greatly lengthened sleep-wake cycle that progressively lengthened over the decade following puberty, combined with profound intellectual disability (ID) and developmental delay. We show that this *GRIA3* mutation alters the conduction pore, rendering the channel more resistant to opening its gate, and that the orthologous mutation in mice also disrupts the organization of sleep and wake bouts.

## Results

### A family with sleep-wake cycle disturbance

**Figure 1:**
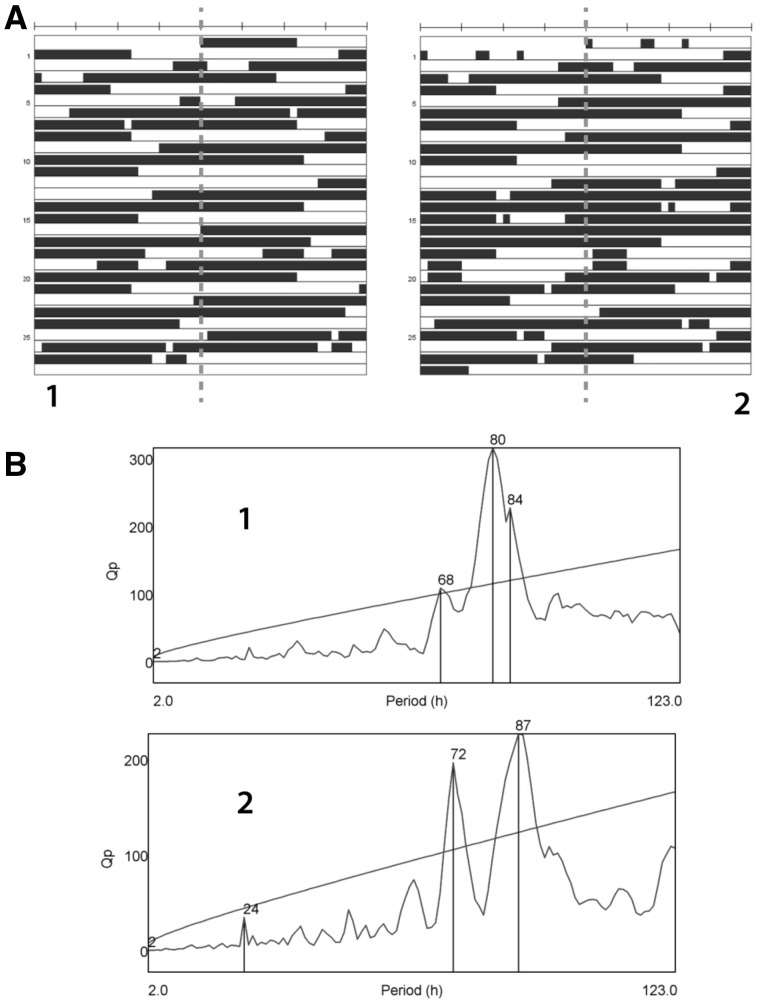
Actograms for two brothers with a *GRIA3*(A653T) mutation. (**A**) Periods of sleep for the two brothers (labelled as 1 and 2 to match periodograms in (B) displayed over 27–28 days, with hourly resolution, displayed as a double-plotted actograms. Each row represents two 24-h periods, with each day plotted twice, first in the second half of one row and then in the first half of the next row. Black indicates periods of reported sleep. The numbers on the Y-axis refer to the days of the record. (**B**) Chi-Square periodograms of sleep from the data in panel A shows multiple peaks at 56-90h, with a smaller peak at 24 h in brother 2 (the younger brother). Qp is the Chi-square statistic for a given period (a measure of power of rhythmicity at that period). The diagonal line represents the level above which results would be considered significant (*P <* 0.01).

A family in which two sons had an undiagnosed, severe developmental disorder was referred for whole-genome sequencing. Their healthy non-consanguineous parents had no family history of developmental delay. The patients had severe ID as well as delayed motor development. They could not run until they were in their teens, and now at 30 and 27, they can still only occasionally speak a few words. Upon reaching puberty, they developed a progressively disturbed sleep-wake cycle, with a gradual prolongation of wake durations. This cycle changed over the course of years with a progressive lengthening over the ten-year period following puberty. In both siblings, this reached extremes of between 60- and 72-h awake, and on one occasion, the older sibling reached 106-h awake, with ensuing sleep of 36 to 48 h. An example of the sleeping patterns is shown in Figure 1. Due to the patients’ limited comprehension, disordered behavior, particularly in unfamiliar settings (e.g. hospitals), and location in a residential home, it was very difficult to perform investigations such as blood tests. Thus, we have very little information on possible underlying alterations in hormone levels, or clinical chemistry, and could not determine, on the basis of the available clinical information, whether their abnormal sleep/wake cycle originates from defects in the circadian system or sleep homeostasis or via a different mechanism.

### Whole genome sequencing identifies a mutation in *GRIA3*

As part of the WGS500 project, a large clinical whole-genome sequencing project in Oxford (23), we sequenced both affected boys and their parents to a coverage of 25X. Only one variant in the WGS data was sufficiently rare, fitted an appropriate inheritance model, and altered the protein sequence (passing our filters described in the Methods). This was a novel non-synonymous single nucleotide variant (SNV) in the X-linked *GRIA3* gene (chrX:122,561,871 G > A) that both brothers inherited from their mother. *GRIA3* encodes GluA3, one of four subunits, which make up tetrameric AMPARs in different combinations (24–26). Missense substitutions and large deletions in this gene have been reported to cause a milder form of ID than seen in our patients, but not sleep disturbances (27–29).

The *GRIA3* mutation, an alanine to threonine change at position 653, was not seen in ∼200,000 chromosomes in the gnomAD database, and nor were any other coding mutations observed in the same exon (exon 12). Indeed, exons 7-15 of GRIA3, which encode the major components of the channel domain, are highly significantly depleted of rare missense variation in the Exome Aggregation Consortium dataset of ∼60,000 exomes (observed/expected = 0.22; *P <* 10^−16^, compared to observed/expected = 0.76 for exons 1–6; *P =* 0.04; Kaitlin Samocha, personal communication). This suggests that mutations in this region are likely to have a deleterious effect.

### The *GRIA3*(A653T) mutation stabilizes the channel in a closed conformation

**Figure 2:**
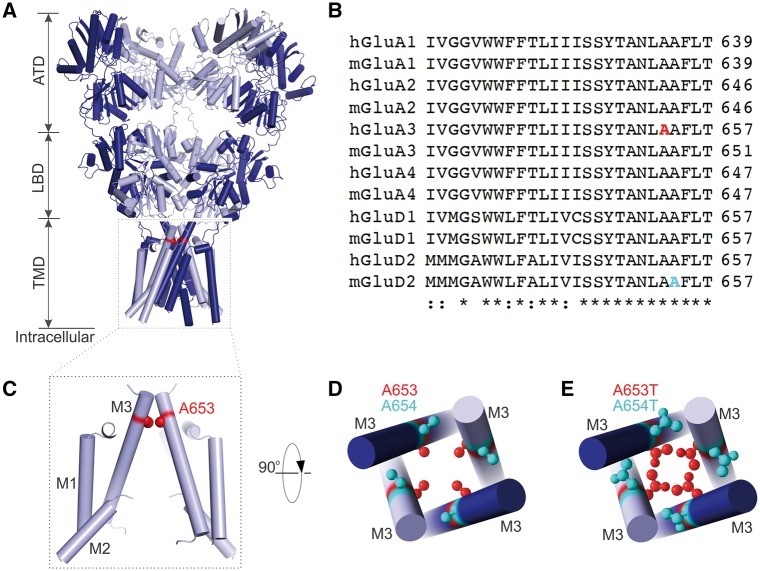
The A653T *GRIA3* mutation relative to the *Lurcher* mutation. (**A**) Structure of a prototypical AMPAR, specifically a GluA2 homotetramer (PDB ID 3KG2) (66). Pore proximal and pore distal subunits are colored in dark and light blue respectively. The layered AMPAR architecture consists of the extracellular amino-terminal domain (ATD) and the ligand binding domain (LBD), followed by the trans-membrane domain (TMD) and intracellular “tails”, disordered and not present in the structure. Residue A621 (3KG2 numbering), equivalent to A653 in GluA3, is colored in red (side chain atoms shown as spheres). (**B**) Sequence alignment of transmembrane helix III (M3) from different human (h) and mouse (m) glutamate receptor proteins. The mutated alanine A653 (red) falls within a highly conserved transmembrane motif that lines the ion channel pore. In GluD2, encoding a different glutamate receptor subunit, the neighbouring alanine residue (cyan) is mutated to threonine in the *Lurcher* mouse (A654T). (**C**) Side view of the four-fold symmetric channel pore, with A653 indicated in red. For clarity, two subunits have been removed. (**D**) Top view of the channel gate, with A653 in red and A654 in cyan. (**E**) Top view of the channel gate, with threonine residues modelled at the alanine sites, to illustrate the potential steric impact of the mutations. While A654T points towards a neighboring subunit, favoring pore opening, A653T leads to pore occlusion.

Ionotropic glutamate receptors have a four-layer domain structure with two extracellular domains, a transmembrane domain that forms the ion channel and a short cytoplasmic tail (Fig. 2A) (26). The A653T mutation alters the receptor in a critical region that forms the channel gate and is highly conserved both across species and in all other ionotropic glutamate receptor genes (30) (Fig. 2B and C). We noted that the neighboring amino acid, A654, is mutated in the orthologous sequence of the GluD2 subunit (formerly GluRδ2) in the “*Lurcher”* mouse (31,32) (Fig. 2B and D), which has been extensively studied as a model of neurodegeneration (33). The Lurcher mutation causes excitotoxicity and apoptotic death of Purkinje cells, which control motor coordination, resulting in the lurching gait of these mice (31). Incidentally, the *Lurcher* is also an alanine to threonine substitution (A654T) and it results in the channel spontaneously opening due to steric hindrance (Fig. 2E). By contrast, the A653T mutation alters a residue projecting towards the ion conduction path, and is therefore expected to stabilize a closed-gate state (Fig. 2E).

**Figure 3:**
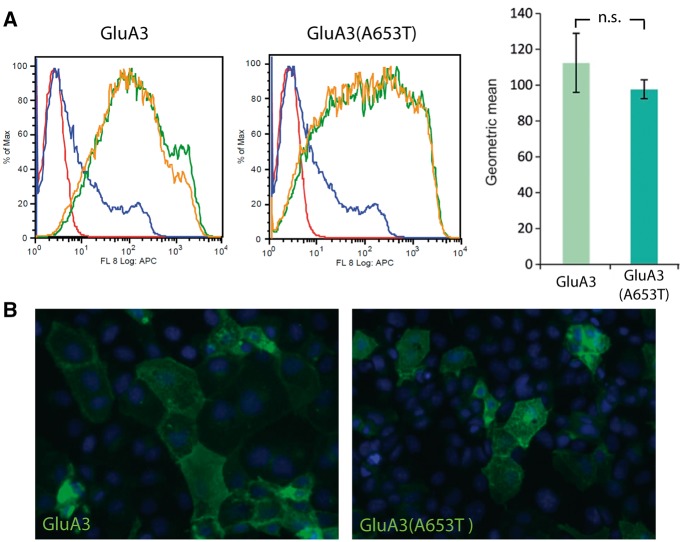
The GluA3 A653T mutation does not interfere with channel trafficking. (**A**) Raw FACS profiles demonstrating membrane localization of GluA3 (left panel) and GluA3(A653T) (right panel) in transfected HEK293T cells (duplicate experiments shown by green and orange traces, untransfected and unstained cells shown by red traces and untransfected and stained cells shown by blue traces). Geometric mean expression values are shown in the histogram and were compared with a *t*-test on the log of the observations; error bars: standard error of the mean (S.E.M.). (**B**) Immunohistochemical staining of Cos7 cells transfected with GluA3 (left panel) and GluA3(A653T) (right panel) showing membrane localization of the receptor.

To test this further, we conducted electrophysiological experiments and assessed the A653T mutation by overexpressing *GRIA3* and *GRIA3*(A653T) cDNA in HEK293T cells. We first established whether the presence of the A653T mutation affected trafficking of the receptor to the membrane. Using HA-tagged GluA3 and GluA3(A653T) constructs, FACS analysis of transfected HEK293T cells revealed no significant alteration in membrane localization of the GluA3(A653T) receptor (Fig. 3A). Furthermore, transfected Cos7 cells also revealed correct membrane localization of the tagged GluA3(A653T) (Fig. 3B).

**Figure 4:**
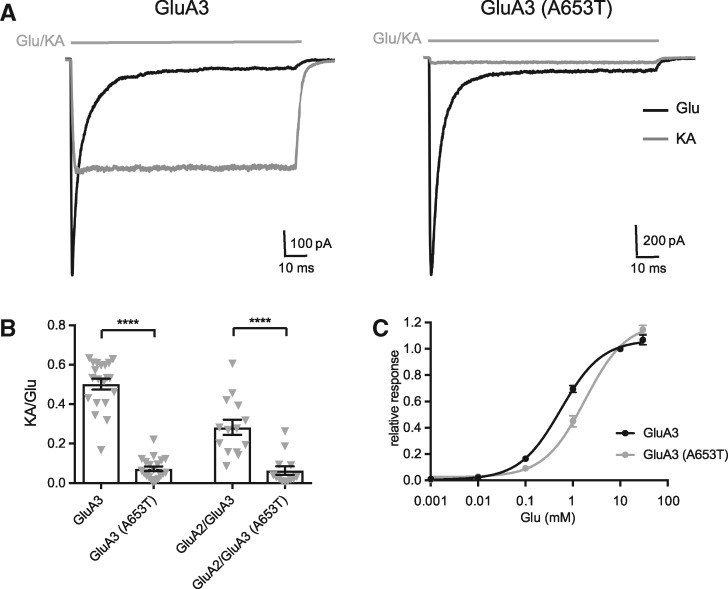
GluA3 with the A653T mutation displays reduced sensitivity to partial agonist kainate. (**A**) Example traces of current responses of GluA3 homomers or GluA3(A653T) homomers; outside-out patches from HEK293T cells stably expressing γ-2, voltage-clamped at -60 mV) to 10 mM l-glutamate (black) and 500 μM kainate (grey). (**B**) Summary graph for recordings shown in (A). The ratio of peak responses to kainate and glutamate (KA/Glu) was 50 ± 7% (*n = *20) and 7 ± 1% (*n = *20) for GluA3 and GluA3(A653T), respectively, and 28 ± 4% (*n = *14) and 6 ± 2% (*n = *13) for GluA2/GluA3 heteromers and GluA2/GluA3(A653T) heteromers, respectively. ****P < 0.0001 (unpaired *t*-test with Welch`s correction). (**C**) Concentration-response characteristics of GluA3 homomers or GluA3(A653T) homomers (in the presence of TARP gamma 2). Peak current responses to 100 ms application of 0.001–30 mM l-glutamate were fitted with a three-parameter dose-response curve (see Methods for details), yielding EC50 values of 0.53 mM and 1.65 mM for GluA3 homomers and GluA3(A653T) homomers, respectively.

When co-expressed with the prototypical AMPAR auxiliary subunit stargazin (γ-2) (to more closely mimic native AMPARs) (34) and when activated by near-saturating concentration of l-glutamate (10 mM), GluA3(A653T) homomers gave current responses with no difference in kinetic properties from GluA3 homomers (black traces in Fig. 4A & data not shown). As expected, GluA3 gave large, virtually non-desensitizing current responses to kainate application (35); however, the activation by kainate was dramatically reduced in GluA3(A653T) (Fig. 4A and B), suggesting that the additional hydrophobic contacts mediated by the threonine at position 653 hamper gate opening.

We further extended this experiment to GluA2/GluA3 heteromers, which are expected to form the main population of GluA3-containing receptors (26,36), and are particularly abundant in the cortex. The same effect on kainate response was observed with GluA2/GluA3(A653T) heteromers, showing that even the presence of only two mutant subunits in a receptor tetramer is sufficient to induce reduced sensitivity to agonists (Fig. 4B). As another measure of agonist efficacy, we constructed a dose-response curve, which revealed that GluA3(A653T) also exhibited a lower sensitivity to glutamate, with a ∼3-times larger EC_50_ for GluA3(A653T) relative to GluA3 (Fig. 4C). Together, these results are in line with the hypothesis that the A653T mutation stabilizes a closed/inactive state of the receptor.

### Mouse model of the A653T mutation

**Figure 5:**
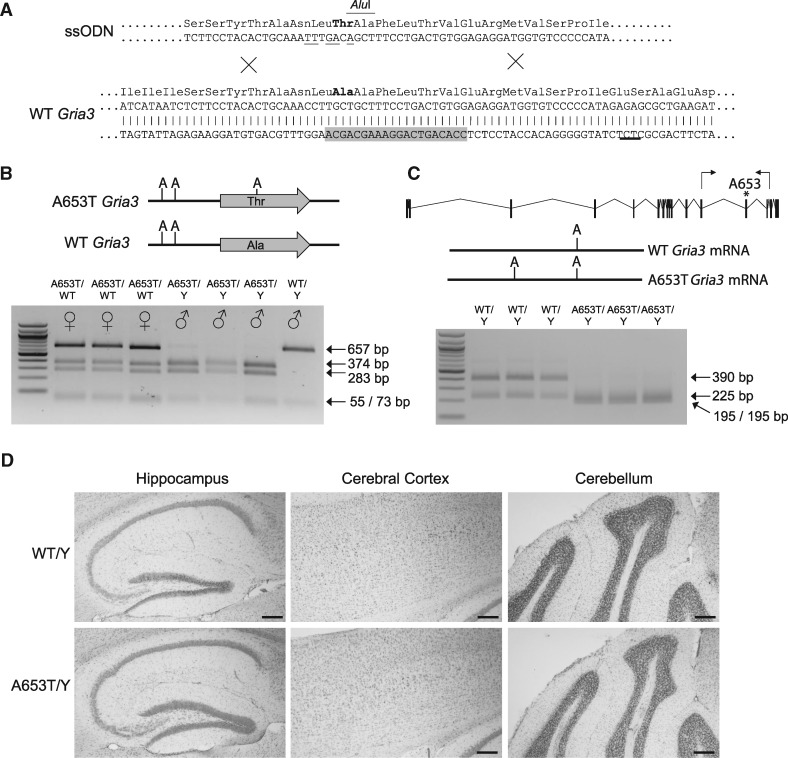
Generation of the *Gria3*^A653T^ mouse model. (**A**) Bottom panel shows the wild-type *Gria3* sequence with the position of the target amino-acid shown in bold. The protospacer target used for the CRISPR-Cas9 nuclease design is highlighted in grey. Top panel shows detail of the ssODN used to introduced the desired amino-acid change, along with silent mutations (underlined) to mark the recombined allele with an AluI restriction site and mutate the Protospacer Adjacent Motif. (**B**) Top panel shows the genotyping amplicon obtained from the *Gria3*^A653T^ and wild-type *Gria3* alleles with the position of the AluI restriction enzymes marker. Lower panel shows an example of the digestion of the amplicon obtained from heterozygous *Gria3*^A653T/WT^ (A653T/WT) female, hemizygous *Gria3*^A653T^ (A653T/Y) and wild-type (WT/Y) male mice. (**C**) Top panel shows the genomic structure of *Gria3* with the position of the primers used for RT-PCR analysis marked. Middle panel shows the pattern of AluI restriction sites in the amplicons obtained from wild-type and A653T cDNA. Lower panel shows the AluI digestion products obtained following ampicon digestion obtained from hemizygous *Gria3*^A653T^ (A653T/Y) and wild-type (WT/Y) male mice. (**D**) Micrographs of Nissl stained sagittal brain sections from wild-type and hemizygous *Gria3*^A653T^ mice in regions of the brain where *Gria3* is highly expressed. Scale bars: 200 μm.

To investigate how the A653T mutation and the predicted reduction in efficacy of the channel could contribute to neurological development, sleep behavior and circadian rhythm, we modeled this mutation in the C57BL/6 mouse. Using CRISPR-Cas9 mutagenesis in ES cells, we introduced the orthologous mutation (A647T) into the *Gria3* gene, and then used these mutated ES cells to generate a line of mice (Fig. 5A and B, Supplementary Material, Fig. S1). We studied a cohort of 12 male litter mates (6 wild-type and 6 with the hemizygous *Gria3*(A653T) mutation, henceforth *Gria3*^A653T^ mice). Hemizygous male mice were physically indistinguishable from their wild-type litter mates and showed no apparent deficits in fertility. Expression of the mutant *Gria3* gene was unaffected by the mutation (Fig. 5C) and gross neuronal architecture was unaltered in the mutant mice (Fig. 5D).

**Figure 6:**
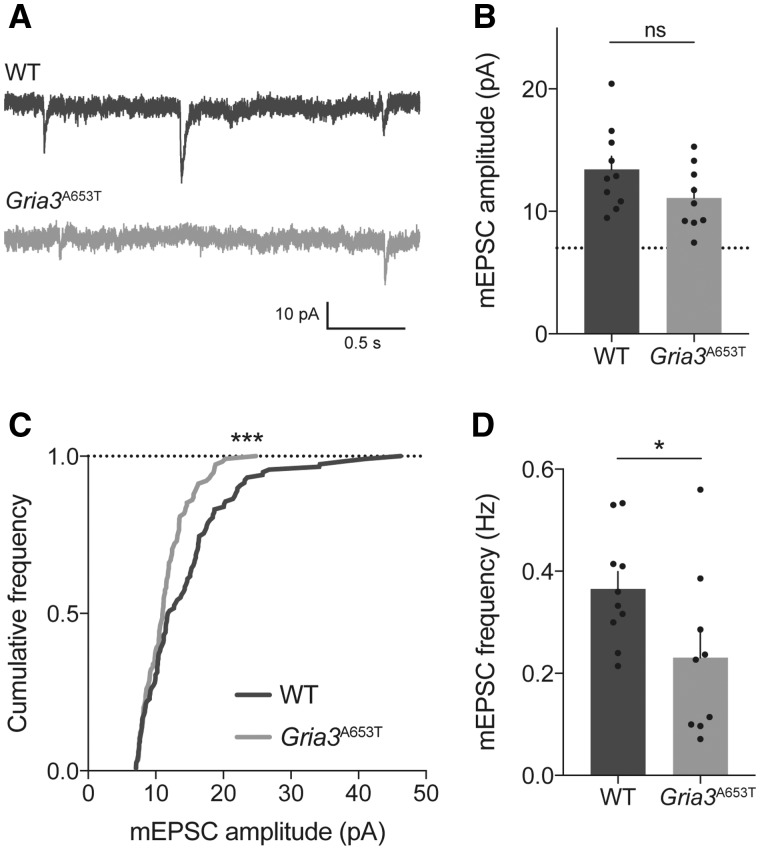
*Gria3* ^A653T^ mice show impaired synaptic transmission. (**A**) Example traces of AMPAR mEPSC recordings from cerebellar Purkinje cells of wild type (WT) and *Gria3*^A653T^ mice. (**B**) Average mEPSC amplitude per cell (WT: 13.44 ± 1.06 (*n = *10 cells); *Gria3*^A653T^: 11.09 ± 0.876 (*n = *9); unpaired *t*-test, *P =* 0.111). (**C**) Cumulative frequency distributions of a representative subset of mEPSC amplitudes show a reduction in large amplitude mEPSCs in *Gria3*^A653T^ mice. Frequency distributions, when fitted with a skewed normal curve (not depicted), are significantly different between populations (F-test, *P =* 0.0001, F(3, 36) = 9.18). (**D**) mEPSC frequency was significantly lower in *Gria3*^A653T^ mice when compared to WT (WT: 0.37 ± 0.03 (*n = *10); *Gria3*^A653T^: 0.23 ± 0.05 (*n = *9); unpaired *t*-test, *P =* 0.0467).

First, to study the effect of the A653T mutation on synaptic transmission, miniature Excitatory Postsynaptic Currents (mEPSCs) were recorded from cerebellar Purkinje neurons (Fig. 6A), which prominently express GluA3 (37). Although the cell-averaged amplitude of mEPSCs was not significantly different between wild-type and *Gria3^A653T^* mice (Fig. 6B; unpaired *t*-test, *P =* 0.111), the distribution of event amplitudes was altered significantly (Fig. 6C;*P =* 0.0001, F_(3,36)_ = 9.18), with an observable decrease in the occurrence of high amplitude mEPSCs in neurons from *Gria3*^A653T^ mice. This finding is in agreement with the data obtained in HEK293 cells (Fig. 4). The frequency of mEPSCs was also reduced in *Gria3*^A653T^ mice when compared to wild-type neurons (Fig. 6D; unpaired *t*-test, *P =* 0.0467), which could be due to a decrease in response amplitude rendering these events undetectable. Hence, synaptic transmission in these model neurons is significantly impaired in *Gria3*^A653T^ mice, likely due to the reduced activation of GluA3(A653T) receptors.

**Figure 7:**
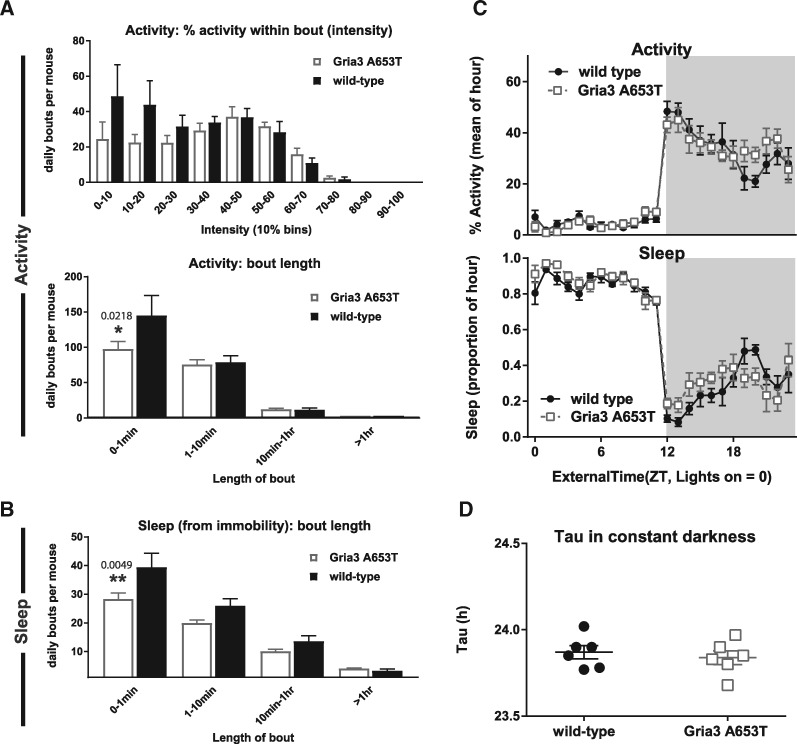
Activity and sleep in *Gria3*^A653T^ mice and wild-type littermates. (**A**) The number of bouts of activity in *Gria3*^A653T^ mice and wild-type (WT) mice (mean ± S.E.M.), separated into the average activity within the bout (intensity, upper panel) and into bout-lengths (lower panel) of <1 min, 1–10 min, 10 min to 1 h and > 1 h (mean ± S.E.M.). *P = 0.0218 for bouts of activity <1 min, 2-way RM ANOVA, with Bonferroni correction. (**B**) The number of bouts of sleep in *Gria3*^A653T^ mice and wild-type littermates, separated into lengths of <1 min, 1–10 min, 10 min to 1 h and > 1 h (mean ± S.E.M.). **P = 0.0049 for sleep bouts <1 min, 2-way RM ANOVA, with Bonferroni correction. (**C**) The 24 h patterns of activity (upper panel) and sleep (hourly mean ± S.E.M.), from 7 days of 12:12 L:D (*n = *6 mice for each genotype in all panels). (**D**) The circadian rhythmicity in constant darkness of wild-type and *Gria3*^A653T^ mice (mean ± S.E.M.).

Typically, mice are polyphasic, exhibiting multiple sleep bouts of sleep several minutes long within a 24-h period, and they are largely nocturnal, with more activity at night and consequently more sleep in the light period of the day (38). Analysis of the bouts of activity and sleep that make up the 24-h patterns revealed differences in the hemizygous *Gria3*^A653T^ mice, against a background of high variability between littermate individuals (as has been reported for wild-type animals previously (39)). Both genotypes showed a similar distribution of the levels of activity within individual bouts, but the *Gria3*^A653T^ mice showed a trend for a decrease in the number of ‘low-intensity’ activity bouts (Fig. 7A, upper panel). *Gria3*^A653T^ mice showed significantly fewer bouts of brief activity (<1 min; *P =* 0.0218 from 2-way RM ANOVA, with Bonferroni correction; Fig. 7A, lower panel) and a similar decrease in brief bouts of sleep (<1 min; *P =* 0.0049, from 2-way RM ANOVA, with Bonferroni correction; Fig. 7B).

No difference was observed between the *Gria3* mutant and wild-type mice in the total daily levels of activity or sleep, or in the 24-h patterns of these measures (Fig. 7C). Both groups of mice showed more activity in the dark (Fig. 7C, upper panel) and a greater proportion of their sleep in the light period (Fig. 7C, lower panel). A repeated measures ANOVA of the 24-h profiles of activity and sleep showed that time (*P <* 0.0001, F_(23, 230)_ =70.05) and individual (*P <* 0.0001, F_(10,230)_ = 11.28) accounted for the majority of variation (81.1% and 5.7%, respectively).

**Figure 8:**
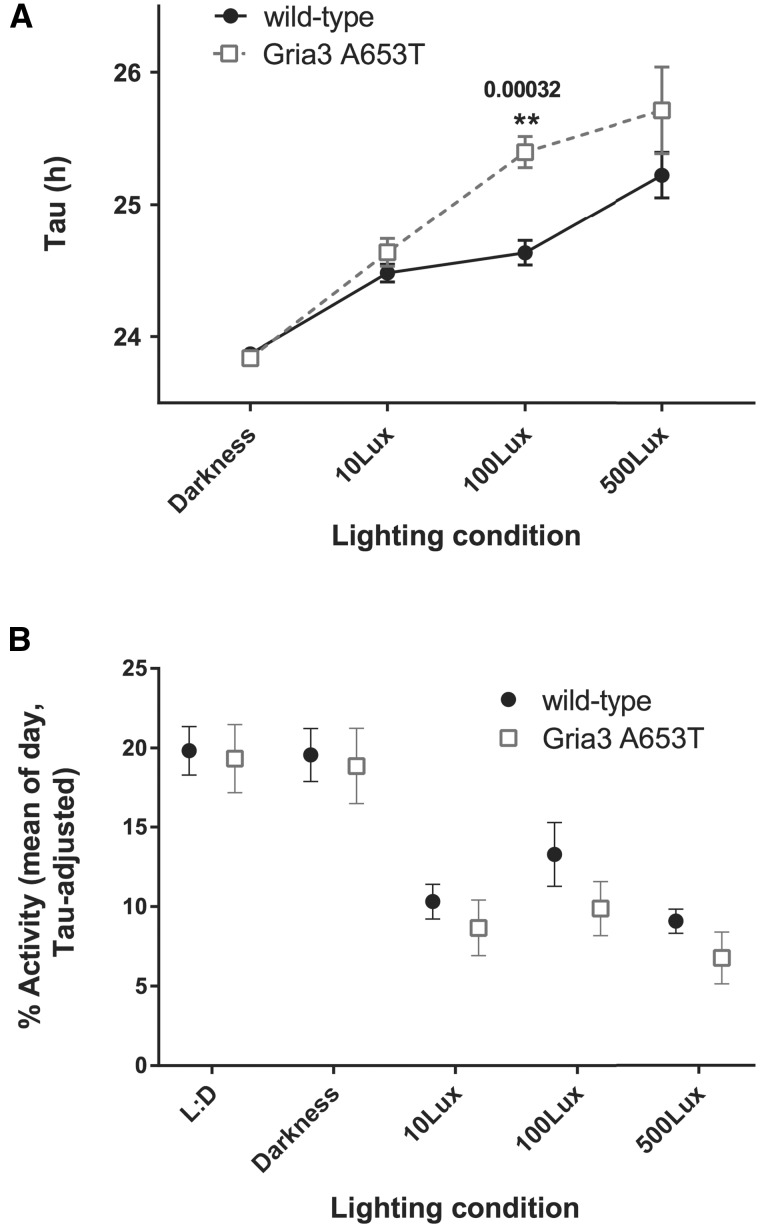
Actigraphy in constant lighting conditions suggests increased sensitivity of the retino-hypothalamic tract in *Gria3*^A653T^ hemizygous male mice. (**A**) Average Tau values from the chi-square periodograms of all mice in constant darkness and three different levels of environmental illumination (mean ± S.E.M.). **P = 0.0032 for 100 Lux, 2-way RM ANOVA, with Bonferroni correction (*n = *6 mice for each genotype). (**B**) Average daily activity levels of all mice in 12:12 Light:Dark, constant darkness and three different levels of environmental illumination (mean ± S.E.M.). Individual values for each mouse were adjusted based on the internal Tau under that lighting condition before averaging (24/Tau x daily average activity). See Supplementary Material, Figure S2 for actograms showing activity patterns.

Although the free-running tau (the timing of the internal clock) in constant darkness was the same in both groups of mice (Fig. 7D), *Gria3^A653T^* mice were more sensitive than wild-type mice to constant light (Fig. 8A). The lengthening of tau with increasing levels of light is expected (40), making up 68.4% of the variation in the data (*P <* 0.0001, F _(3, 30)_ = 44.1, for light level in a 2-way RM ANOVA), but the effect of genotype was also significant, explaining 5.73% (*P =* 0.0107, F _(1, 10)_ = 9.79). This suggests an increased sensitivity of the retino-hypothalamic tract to light in *Gria3*^A653T^ mice. However, any changes in sensitivity to light did not lead to increased suppression of activity (masking) in the *Gria3^A653T^* mice, when compared to their wild-type littermates (Fig. 8B). Constant light at all intensities suppressed daily levels of activity (with lighting accounting for 60.2% of variation in the data, *P <* 0.0001, F (4, 40) = 102.6, in a 2-way RM ANOVA), whilst genotype was not a significant factor (1.8% of variation in the data, *P =* 0.4619, F (1,10) = 0.5853).

Overall, in standard light conditions, the *Gria3^A653T^* mice exhibited differences in the structure of bouts of sleep and activity when compared to their wild-type littermates, even though their circadian rhythms remained robust. When active, the *Gria3*^A653T^ mice were less likely to display short, low-intensity bouts of activity and, when sleeping, they were less likely to have short bouts of sleep. Furthermore, in constant light, their tau lengthened to a greater extent than that of wild-type mice.

## Discussion

We have discovered a mutation in the known X-linked ID gene *GRIA3* that causes severe sleep-wake cycle disturbances in two brothers. This mutation is situated at the neighbouring amino acid to the orthologous one mutated in a mouse neurobehavioral mutant, the *Lurcher* mouse, and involves the same two amino acids: an alanine to threonine substitution. The *Lurcher* mutation causes spontaneous opening of the GluD2 ion channel, leading to excitotoxicity and eventual neurodegeneration of cerebellar Purkinje cells, where it is predominantly expressed. (No sleep phenotype has been reported.) Based on its position (Fig. 2E), we hypothesized that the A653T mutation would stabilize the channel in a closed-gate state, and that this could lead to pathologies in the parts of the brain in which GluA3 is expressed, potentially explaining the clinical phenotype.

To investigate its effect *in vivo*, we introduced the orthologous A653T mutation into mice. We anticipated that the phenotype might be quite subtle, like that of the *Gria3* knock-out mice. Steenland, Kim and Zhuo (41) did not observe circadian rhythm disturbances in the *Gria3* knock-outs, but they did find that mutants lack electroencephalographic signatures of NREM sleep (41). The *Gria3* knock-out mice have essentially normal hippocampal synaptic function, with a notable increase in hippocampal long-term potentiation (42), and have been reported to show motor learning deficits (37), increased social and aggressive behavior (43), and alterations in exploratory behavior (44). *Gria3* knock-out mice also show deficits in cerebellar Purkinje cell synaptic transmission, indicating that a substantial population of synaptic receptors contain GluA3 (37). *Gria3*^A653T^ mice show similar deficits, with reduced amplitude and frequency of spontaneous synaptic AMPAR currents (Fig. 6), reflecting impaired receptor activation, as seen in recordings obtained from recombinant GluA3 receptors (Fig. 4). These data confirm that the GluA3(A653T) mutation causes deficits in AMPAR activation in a synaptic context. Whether this deficit could explain elements of the patient’s phenotype remains unclear, but it seems reasonable to assume that synaptic function may be similarly impaired in other areas of the brain where GluA3 is expressed. With respect to cerebellar synaptic function, we cannot rule out that deficits in synaptic transmission to Purkinje cells may contribute to the motor function delay of the patients, but it is worth noting that no obvious motor deficits were observed in the *Gria3*^A653T^ mice, although specific tests were not performed. Intriguingly, impaired cerebellar function has been linked to sleep deficits (reviewed in (45)) which might point to a direct link between the impaired cerebellar synaptic transmission and the sleep phenotype observed.

We investigated circadian rhythms and behaviorally-defined sleep in the *Gria3*^A653T^ mice. Given that the structure of sleep differs so markedly between humans and mice, we did not expect to see a phenocopy of the human mutation, and indeed, the dramatic changes in sleep-wake timing reported in the patients were not recapitulated in the *Gria3*^A653T^ mice. However, whilst circadian rhythms in locomotor activity and behaviorally-defined sleep were relatively normal in mutant mice, we did observe a significant decrease in the number of short (<1 min) bouts of both activity and sleep, suggesting that the underlying systems regulating sleep and activity may have undergone subtle changes in the mutant. In addition, these mice displayed increased sensitivity to light, suggestive of an alteration in retino-hypothalamic response, which may underlie the behavioural differences observed. Indeed, AMPAR subunits are known to play a role in retinal ganglion cells (46–48). Furthermore, it has been shown that aniracetam, an AMPAR agonist, potentiates photic responses of the biological clock in rodents (49), suggesting a role for AMPARs in light input to the SCN. It is important to note that the increased sensitivity to constant light in the *Gria3^A653T^* mice did not lead to an increased suppression of activity (masking). The effects of light on period length but not activity suppression suggest that the effects of light may be upon the retina or SCN, rather than at the level of the subparaventricular zone (SPVz), which has been implicated in mediating masking responses (50). This is in contrast to recent studies on a mouse model of Smith-Magenis Syndrome, in which retinal and SCN responses were attenuated, but SPVz responses were enhanced (51).

Thus, although the *Gria3*^A653T^ mutation does not change the circadian period or the 24-h profile of activity or sleep under normal conditions, it does change the distribution of sleep and activity bouts in mice. This suggests that the *GRIA3* mutation in the human patients may be affecting the organization of the sleep-wake cycle rather than circadian rhythms, which was not clear from the initial clinical observations.

There could be several reasons why the human phenotype resulting from this mutation appears much more extreme than that in mice. One could be that the spatial or temporal expression pattern of the GluA3 subunit differs between humans and mice, as it does between mice and other species. In mice, GluA3 is the predominant AMPAR subunit in the thalamus (52), which plays an important role in the sleep-wake cycle (53), but it is expressed at very low levels or not at all in the SCN (54). In contrast, in rats and monkeys, GluA3 is expressed at high levels in the SCN (55), and, in rats, it is also expressed in the pineal gland, which regulates melatonin production (56). The expression or activity of GluA3 in humans may be dependent on diurnal activity, while mice are typically nocturnal. It could also be that the relative expression of the four different AMPAR subunits differs between species, altering channel conductance properties and gating kinetics, and leading to differences in phenotypic consequence.

It is unclear why our patients with the *GRIA3*^A653T^ mutation have such severe disturbances in sleep-wake timing, while a large deletion and other missense mutations, including one lying within a transmembrane domain (G833R), apparently cause ID without sleep-wake cycle disturbances (29,57) (or at least, these were not reported). It may be that the large deletion and the G833R mutation result in a different outcome to A653T because they result in reduced GluA3 expression (29), which may be compensated for by the other AMPAR subunits, whereas the A653T mutation does not influence trafficking of GluA3 (Fig. 3), so should not change the stoichiometry of the receptors.

In conclusion, we have identified a mutation in the known ID gene *GRIA3* that causes severe sleep-wake cycle dysregulation in two human brothers. Gene-edited mice harboring this mutation showed subtle changes in the structure of sleep and activity, and an increased sensitivity to constant light, but their 24-h rhythm was otherwise unchanged under normal light/dark conditions. Our results point to an important role for *GRIA3* in regulating the sleep-wake cycle in both species.

## Materials and Methods

### Clinical description

The patients, two brothers, now 30 and 27 years of age, were born 3 years apart to healthy, unrelated parents. Both showed significant developmental delay. The older brother was born at 38 weeks’ gestation and was severely hypotonic at birth, and had significant feeding problems. He developed head control at 6 months, stood with support at age 3 years, and walked at age 3.5. The younger brother was not hypotonic at birth, but truncal hypotonia was noted at 6 months. He sat at age 3–4 years, stood at age 6 and walked at age 7. Both have severe ID with expressive language limited to a few words, and limited receptive language. A significant degree of ataxia and dyspraxia became apparent as they aged, though they were both able to feed themselves with assistance, dress with a high level of support and walk unaided. Their sleep-wake cycle was normal until each entered puberty at about 13 years, after which a change in circadian behavior developed. This was characterized by a progressive elongation of sleep-wake cycles over the next decade. The sleep-wake disturbance was not improved by treatment with melatonin 2 mg given nocte, and it often had to be aborted (due to increasing agitation in the patients) by a benzodiazepine (diazepam), in combination with a sedating drug such as quetiapine and olanzapine, given after 40 or more hours being awake. The patients underwent numerous pharmacological treatments, none of which had a substantial impact on their condition. The parents gave consent for clinical genome sequencing as part of the WGS500 project (23).

### Actograms from sleep diaries

Sleep diaries were recorded for both brothers, with hourly assessment over 27–28 days, when the brothers were 24 and 21 years of age. These sleep diaries were coded in the first instance to differentiate sleep (sleepy and rousable) from all waking states (from quiet waking to hyperactivity). The hourly patterns of sleep were then used to generate double-plotted actograms and chi-square periodograms in the ActogramJ plugin (version 0.9, http://actogramj.neurofly.de/; date last accessed December 1, 2016) of the ImageJ program (version 1.5.0e, http://imagej.nih.gov/ij/; date last accessed December 1, 2016) (Fig. 1).

### Whole-genome sequencing and variant filtering

DNA was extracted from saliva and then sequencing was carried out as described in (23). WGS was conducted on the Illumina HiSeq platform to a coverage of at least 25X. The reads were mapped to the human reference genome (build 37d5) with Stampy (58) and single nucleotide variants (SNVs) and small indels were called with Platypus (59). Several methods were used to search for copy number variants (CNVs) (see (23)). Variants were annotated relative to RefSeq transcripts using ANNOVAR (60) and relative to Ensembl transcripts using an in-house tool called VariantAnno (https://bitbucket.org/humburg/variantanno; date last accessed January 5, 2012), similar to the Ensembl Variant Effect Predictor.

We focused on variants that were predicted to alter the protein sequence (nonsynonymous SNVs, stop loss or gain variants, indels, or splice site mutations). We first considered the simple, compound and X-linked recessive models, which seemed most likely given that both brothers were affected. We excluded variants with a frequency greater than 0.005 in the 1000 Genomes or the NHLBI Exome Sequencing Project (ESP), or for which there were any homozygotes or hemizygotes or more than five heterozygotes amongst the other 294 WGS500 samples available at the time of analysis. Then, post-hoc, we excluded any variants seen as homozygotes or hemizygotes in gnomAD, a database of 126,216 exome sequences and 15,136 whole-genome sequences from unrelated individuals without severe paediatric disease (http://gnomad.broadinstitute.org/; date last accessed January 31, 2017). For the compound heterozygous model, we required two such variants in the same gene, one inherited from each heterozygous parent.

We also considered the possibility that both patients had inherited the same *de novo* mutation from one parent. To check for this, we searched for variants that were heterozygous in both brothers but homozygous reference in both parents, with the genotype log likelihood ratio less than -5 in all individuals. When we examined these candidate *de novo* variants in the Integrated Genomics Viewer (61), all cases appeared to be artefactual or due to incorrect calling of the parental genotype.

### Structural analysis of the A653T mutation

Structural panels in Figure 2 were prepared with the PyMOL Molecular Graphics System, Version 1.8, Schrödinger, LLC. We used a model of the GluA2 homotetramer (PDB ID: 3KG2) rather than of the GluA2/A3 heterodimers, because this is the best model of an AMPAR available, and the pore region is very similar between them.

### Membrane localization analysis

HEK293T cells were transfected with HA-tagged rat GluA3 and GluA3(A653T) cDNA expression constructs harboring the R461G (“flip”) mutation to increase cell surface expression (62) (Uniprot accession number P19492-2, residues F24-I888) using Lipofectamine ™ 2000 reagent (ThermoFisher Scientific). Cells, together with mock transfected control cells, were collected and washed 2x with ice-cold PBS containing 0.05% sodium azide (PBS/NaN_3_), prior to incubation for 45 min with primary mouse anti-HA antibody (Sigma H3663; 0.5 ng/µl in 100 µl PBS/NaN_3_) at 4 °C. After 3x washing in PBS/NaN_3_, cells were incubated for 30 min with secondary donkey anti-mouse antibody, conjugated to APC (ThermoFisher Scientific) (0.5 μg/ml in 100 µl PBS/NaN_3_) at 4 °C in the dark. Cells were washed 3x as before and resuspended in 2% paraformaldehyde in PBS/NaN_3_. Cells were sorted using a CyAn cell sorter (Beckmann Coulter). Geometric mean expression values were calculated from replicate experiments using the FlowJo software package (FlowJo LLC).

For imaging of membrane localization, Cos7 cells were transfected with HA-tagged rat GluA3 and GluA3(A653T) cDNA expression constructs as above. Cells were fixed in 4% paraformaldehyde/PBS, blocked in 1% bovine serum albumin(BSA)/PBS before incubation with mouse anti-HA antibody (Sigma H3663; 1:1000 in 3% BSA/PBS). After washing 3x in 0.1% BSA/PBS, cells were stained with fluorescently labeled secondary goat anti-mouse antibody conjugated to Alexa488 Fluor® (Abcam), washed 3x, rinsed in water and mounted with Fluorosave (Millipore) containing a nuclear DAPI stain. Stained cells were visualized using a Zeiss 510 MetaHead confocal fluorescence microscope.

### Animal work

The generation and phenotyping of the *Gria3*^A653T^ mouse model was carried out in accordance with Animal [Scientific Procedures] Act 1986, with procedures reviewed by the clinical medicine animal care and ethical review body (AWERB), and conducted under project licenses PPL30/2966, PPL 30/2812 and PPL 70/8135. Animals were housed in specific pathogen free conditions, with the only reported positives on health screening over the entire time course of these studies being for *Helicobacter hepaticus* and *Entamoeba* spp. All animals were singly-housed, provided with food and water ad-libitum and, maintained on a 12h light:12h dark cycle (150–200 lux cool white LED light, measured at the cage floor), or, during experimental conditions, in light-tight environmental enclosures (in 2 groups of 6 cages). Phenotyping experiments were not blinded or randomized but wild-type and mutant animals were alternated between experiments. All environmental changes were applied to all animals and no animals were excluded from the study.

### Generation of the *Gria3^A653T^* mouse

The target amino-acid residue, A653 in the human *GRIA3* cDNA is equivalent to A647 in the mouse *Gria3* cDNA. For greater clarity, we refer to the allele and the mouse mutant as *Gria3^A653T^*throughout the manuscript. A target site for CRISPR-Cas9 mutagenesis (5’-CCACAGTCAGGAAAGCAGCA-3’) was identified in the region of exon 12 of the mouse *Gria3* gene, close to the Alanine-647 codon (orthologous to the A653 in the human *GRIA3* cDNA) using the MIT CRISPR design tool (crispr.mit.edu). The selected target protospacer was validated by direct zygote injection and high rates of mutagenesis were observed with no evidence of off-target mutagenesis (63). This target site protospacer was cloned into the BbsI restriction sites within pX330 (Addgene Plasmid #42230), modified with the addition of a puromycin resistance cassette, as a linker formed by annealing two oligonucleotides (5’-CACCGCCACAGTCAGGAAAGCAGCA-3’ and 5’-AAACTGCTGCTTTCCTGACTGTGGC-3’), generating plasmid pX330-Puro-Gria3. A 139 nucleotide single-stranded oligonucleotide (ssODN) template (5’- CAGTCTGCTTGGCTAAATCTTCAGCGCTCTCTATGGGGGACACCATCCTCTCCACAGTCAGGAAAGCTGTCAAATTTGCAGTGTAGGAAGAGATTATGATCAGGGTGAAGAACCACCAAACCCCTCCAACAATGCGCCC-3’) was synthesized (Eurogentec), which harbored the desired A647T mutation along with a novel AluI restriction site, incorporated into the sequence as silent mutations. These silent mutations also removed the Protospacer Adjacent Motif downstream of the CRISPR-Cas9 targeting site, thus rendering the recombined allele resistant to further mutagenesis once homology directed repair had occurred.

1×10^6^ JM8F6 embryonic stem (ES) cells (C57BL/6N, a kind gift from the Wellcome Trust Sanger Institute and tested mycoplasma free) were electroporated with 5 µg of the cloned pX330-Puro-Gria3 plasmid and 2 µg of the ssODN using the Neon Transfection System (Thermo Fisher Scientific) (3 × 1400 V, 10 ms) and plated on Puromycin resistant fibroblast feeder layers. After approximately 24 h, selection in 600 ng/ml puromycin was applied for a further 48 h to allow transient selection. After a further 5 days in culture without selection, individual colonies were isolated, expanded and screened for the desired mutagenesis event using specific primers (5’-AACTAGAGAAAACCTGGAGAGGCC-3’ and 5’-CCCGTGAGTCTAATGGACAATGGA-3’) to amplify a region of *Gria3* exon 12, followed by AluI digestion to detect incorporation of the template sequence. The presence of the A647T mutation was confirmed by Sanger sequencing in four independent ES cell clones, from a total of 82 screened, representing a targeting frequency of approximately 5%.

ES cells from a single correctly targeted clone were injected into albino C57BL/6J blastocysts and 5 chimeras with over 70% ES cell contribution were generated. Two of the resulting chimeras were mated with albino C57BL/6J females and successful germline transmission was confirmed for one of these chimera breedings. The presence of the mutation in the F1 generation was confirmed at the DNA level by the above PCR and Sanger sequencing. Mouse colonies were maintained by backcrossing to C57BL/6J mice. The gene-edited mouse model described in this manuscript has been attributed the allele name *Gria3^em1Wthg^* (MGI Accession ID 5898442).

### Expression analysis

Expression of the *Gria3* alleles was investigated by endpoint RT-PCR on cDNA prepared from 1 µg of cortex RNA (ReliaPrep RNA tissue miniprep kit, GoScript Reverse Transcription System, Promega), using a forward primer (5’-AACCTCGTGACCCACAAAG-3’) binding in exon 11 and a reverse primer (5’-GCTTGTCTAAGATGCCTTGTTC-3’) binding in exon 14, thus spanning the introduced A653T mutation. Digestion of the RT-PCR product with AluI was used to distinguish the mutant A643T transcript from the wild-type transcript.

### Electrophysiology

*Recombinant receptor recordings* *.* HEK293T cells (ATCC) stably expressing the prototypic AMPAR auxiliary subunit TARP gamma 2 were transfected either with GluA3 (the “flip” isoform) or a combination of GluA3 and GluA2 (flop, R) expression plasmids using the Effectene® transfection reagent (Qiagen). When co-expressing GluA2 with GluA3, the formation of heteromers was checked for each patch by recording the I/V characteristics of the responses in the presence of intracellular spermine. Following transfection, to protect from AMPAR-mediated toxicity, the cells were grown in the presence of 30 μM 2,3-dioxo-6-nitro-1,2,3,4-tetrahydrobenzo[f]quinoxaline-7-sulfonamide (NBQX; Tocris-ABCam).

Current responses of outside-out patches excised from the transfected cells and voltage-clamped at −60 mV were elicited by fast application of 10 mM l-glutamate or 500 μM kainate via a Θ-tube and recorded using an Axopatch-1D amplifier, a Digidata1322 interface and pClamp 9.2 software (Molecular Devices). Cells were perfused with external solution containing (in mM): NaCl (145), KCl (3), CaCl_2_ (2), MgCl_2_ (1), glucose (10) and HEPES (10), adjusted to pH 7.4 with NaOH. Electrodes were fabricated from borosilicate glass (1.5 mm o.d., 0.86 mm i.d., Science Products GmbH) pulled with a PC-10 vertical puller (Narishige). When filled with the ‘internal’ solution, containing (mM): CsF (120), CsCl (10), EGTA (10), ATP-sodium salt (2), HEPES (10) and spermine (0.1), adjusted to pH 7.3 with CsOH, they had a final resistance of 2–5 MΩ.

For the dose-response curve the outside-out patches were activated by different concentrations of l-glutamate, ranging from 1 μM to 30 mM, and the current amplitudes were normalized to the currents elicited by 10 mM l-glutamate. The relative response values were then plotted against the agonist concentration using the three parameter dose-response curve in Prism 6.0 (GraphPad Software): Y, where Y is the relative response and X is log[l-glutamate].

*Slice* *e* *lectrophysiology* *.* Mice were anaesthetised using an overdose of sodium pentobarbital. Acute, 300 μm thick, sagittal cerebellar slices were cut from 8- to 10-week-old mice in ice cold solution containing (in mM): 93 NMDG, 93 HCl, 2.5 KCl, 1.2 NaH_2_PO_4_, 30 NaHCO_3_, 20 HEPES, 25 Glucose, 5 Na ascorbate, 2 thiourea, 3 Na pyruvate, 5 N-acetyl-L-cysteine, 10 MgSO_4_, 0.5 CaCl_2_, pH 7.3. Slices were recovered for 15 mins in the above solution at 37 °C before incubation in artificial cerebrospinal fluid (aCSF) at 37 °C prior to recording. aCSF contained (in mM): 125 NaCl, 2.5 KCl, 1.25 NaH_2_PO_4_, 25 NaHCO_3_, 10 glucose, 1 Na pyruvate, 4 CaCl_2_, 4 MgCl_2_ at pH 7.3 and saturated with 95% O_2_/5% CO_2_. 0.001 SR-95531, 100 μM D-APV and 1 μM tetrodotoxin were used to isolate AMPAR mEPSCs and recordings were performed at 25 °C. 3–6 MΩ borosilicate pipettes were filled with intracellular solution containing (in mM): 135 CH_3_SO_3_H, 135 CsOH, 4 NaCl, 2 MgCl_2_, 10 HEPES, 4 Na_2_-ATP, 0.4 Na-GTP, 0.15 spermine, 0.6 EGTA, 0.1 CaCl_2_, at pH 7.25. Purkinje neurons were voltage-clamped in the whole-cell configuration at a holding potential of −60 mV. Recordings were collected using a Multiclamp 700B amplifier (Axon Instruments) and digitized at 100 kHz with series resistance monitored using a −10 mV holding potential step, repeated at 10 s intervals. Recordings in which the series resistance varied by more than 20% were discarded. mEPSC analysis was conducted as described previously (64). mEPSC detection was conducted using a template-based search in Clampfit (Molecular Devices). To limit the influence of dendritic filtering, mEPSCs with a rise-time slower than 2 ms were excluded for comparison of event amplitude. Cumulative frequency distributions were plotted using equal sized samples of mEPSCs from all recorded cells.

### Circadian and sleep phenotyping

Experiments were designed based upon an alpha of 0.05 and power of 0.80. Based on previous phenotyping data from studies on circadian locomotor activity, given an expected coefficient of variance of 30%, a sample size of 6 was used as this would enable us to resolve a 50% change in activity levels with a power of 0.82. Accordingly, a cohort of 12 male littermate mice was used to study activity and sleep phenotypes (6 wild-type and 6 with the hemizygous A653T *Gria3* mutation). All experiments were carried out when the mice were at least 6 weeks of age (i.e. after sexual maturity, when the human patients began to manifest the sleep phenotype). Periods of both constant darkness (<1 lux) and constant light (10, 100 and 500 lux) were used to establish circadian parameters, with a minimum uninterrupted period of at least 9 days for each lighting condition. Analysis of activity and sleep was carried out between groups and no mice were excluded from any group in this work.

Activity data were recorded using passive infrared sensors, with polling every 100ms (% time active reported at 10s intervals, as described by Brown, Hasan, Foster and Peirson (65)). Activity data were analysed and scored for sleep based on immobility of ≥ 40s using the python PyData stack (http://pydata.org/; date last accessed December 1, 2016), including the Pandas library (version 0.18.0), the Seaborn package (http://seaborn.pydata.org/; date last accessed December 1, 2016, version 0.7.0) and Matplotlib (http://matplotlib.org/; date last accessed December 1, 2016, 1.5.1). Circadian characteristics of the activity were established by exporting activity data as .csv files into ActogramJ for plotting of actograms and generation of chi-square periodograms (as described above in the section on the sleep diaries from the patients).

Seven days of activity and sleep under a standard 12:12 light:dark (L:D) cycle were also analysed for bouts of activity and sleep. The passive infrared sensor used to measure the movement of a mouse was read every 100 ms and reported as percentage activity every 10 s. A bout of activity was considered as one or more consecutive 10 s bins in which the mouse was active. The intensity of activity in the bout is the percentage of the entire bout that the sensor is active. For example, six consecutive bins with activity would give a bout length of 1 min and a 50% intensity would mean the sensor was active for 30 s of that 1 min. If the sensor detected no movement for 4 consecutive bins, a bout of sleep was recorded (starting with the fourth 10 s bin), as 40 s of immobility has been shown to correlate highly with sleep in mice. Further statistical analysis was carried out using GraphPad Prism (version 7, GraphPad software Inc.). Equal variance was assumed based on our observations, and in cases in which the data was not clearly normally distributed, we still considered ANOVA a justified method of analysis due to the large number of bouts.

To generate Figure 5D, neuronal architecture was assessed using 20 μm saggital cryosections and counter staining with cresyl violet.

## Supplementary Material

Supplementary Material is available at *HMG* online.

## Supplementary Material

Supplementary Figure S1

Supplementary Figure S2

Supplementary File 1
