# Supplementary figures and images for: A point mutation in the ion conduction pore of AMPA receptor GRIA3 causes dramatically perturbed sleep patterns as well as intellectual disability
Source: Hum Mol Genet. 2017 Jul 14;26(20):3869–82. doi: 10.1093/hmg/ddx270 (PMC5639461; doi:10.1093/hmg/ddx270)

# Supplementary Figure 1

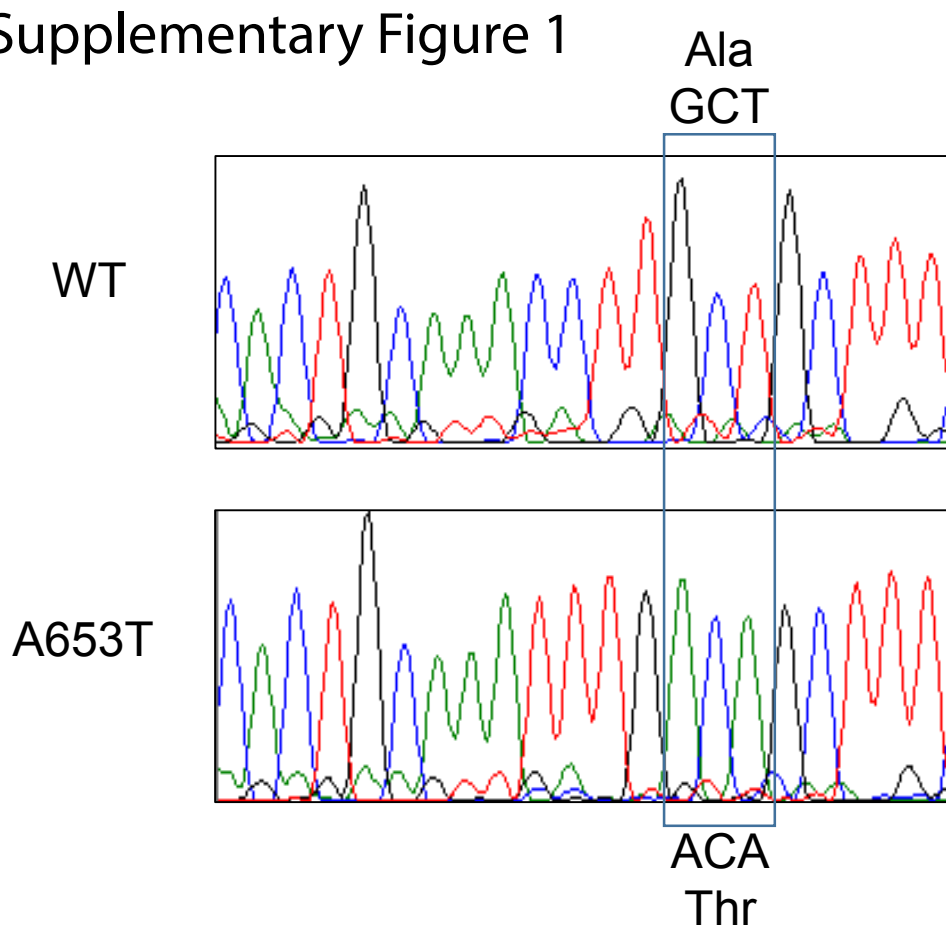

Supplement: Supplementary Figure S1 [file Supplementary_Figure_1.labeled_ddx270.pdf]

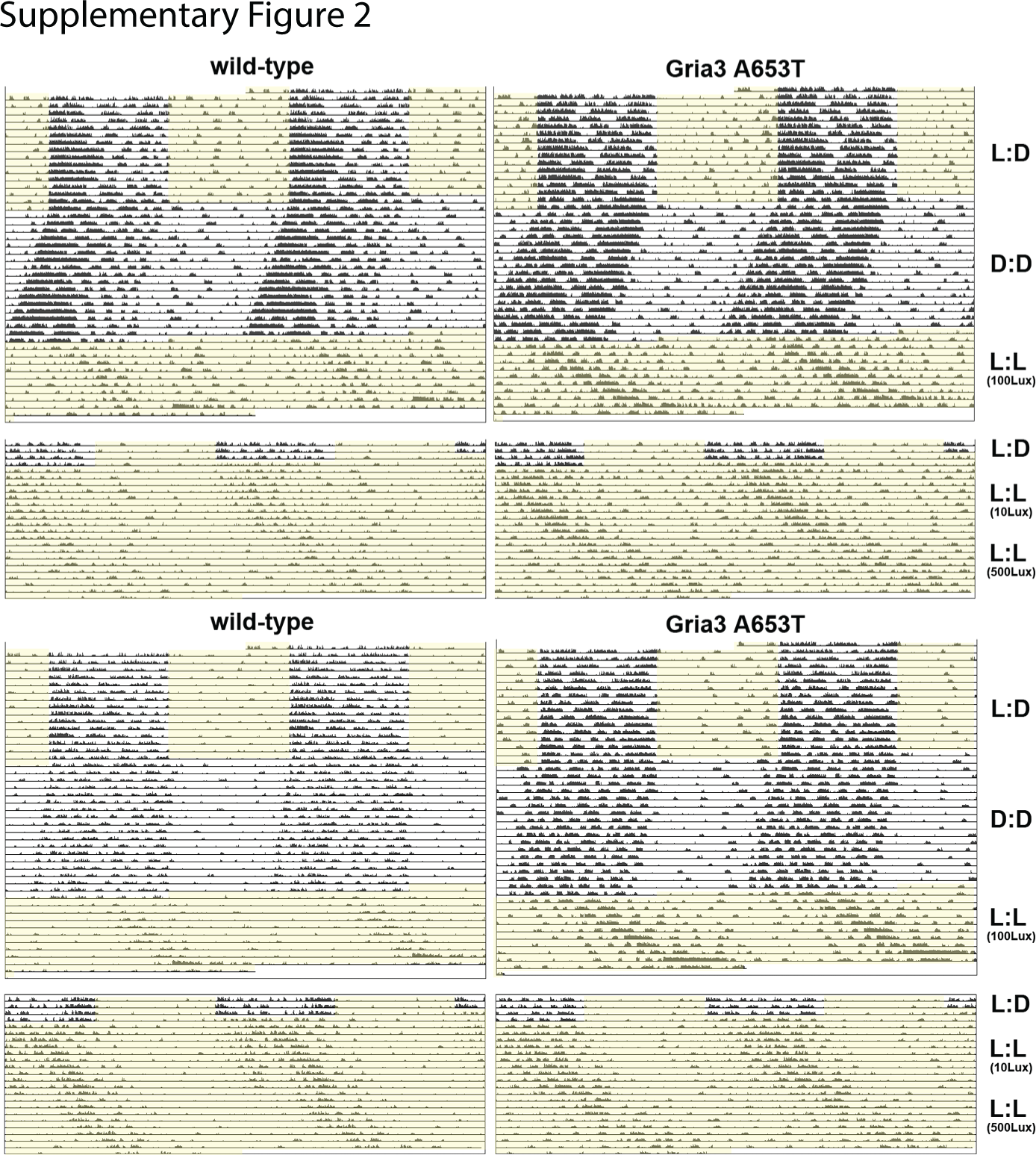

Supplement: Supplementary Figure S2 [file Supplementary_Figure_2.labeled_ddx270.png]
