# Supplementary material for: A point mutation in the ion conduction pore of AMPA receptor GRIA3 causes dramatically perturbed sleep patterns as well as intellectual disability
Source: Hum Mol Genet. 2017 Jul 14;26(20):3869–82. doi: 10.1093/hmg/ddx270 (PMC5639461; doi:10.1093/hmg/ddx270)
Supplement: Supplementary File 1 [file Supplementary_File1_WGS500_authors_ddx270.docx]

**Supplementary File 1**

List of WGS500 Consortium members

Steering Committee

Peter Donnelly (Chair)^1^, John Bell^2^, David Bentley^3^, Gil McVean^1^, Peter Ratcliffe^1^, Jenny Taylor^1,4^, Andrew Wilkie^4, 5^

Operations Committee

Peter Donnelly^1^ (Chair) John Broxholme^1^, David Buck^1^, Jean-Baptiste Cazier^7^, Richard Cornall^1^, Lorna Gregory^1^, Julian Knight^1^, Gerton Lunter^1^, Gilean McVean^1^, Jenny Taylor^1,4^, Ian Tomlinson^1, 4^, Andrew Wilkie^4, 5^

Sequencing & Experimental Follow up

David Buck^1^ (Lead), Christopher Allan^1^, Moustafa Attar^1^, Angie Green^1^, Lorna Gregory^1^, Sean Humphray^3^, Zoya Kingsbury^3^, Sarah Lamble^1^, Lorne Lonie^1^, Alistair Pagnamenta^1^, Paolo Piazza^15^, Guadelupe Polanco^16^, Amy Trebes^1^

Data Analysis

Gil McVean^1^ (Lead), Peter Donnelly^1^, Jean-Baptiste Cazier^1^, John Broxholme^1^, Richard Copley^8^, Simon Fiddy^9^, Russell Grocock^3^, Edouard Hatton^1^, Chris Holmes^1^, Linda Hughes^1^, Peter Humburg^1^, Alexander Kanapin^10^, Stefano Lise^11^, Gerton Lunter^1^, Hilary Martin^12^, Lisa Murray^3^, Davis McCarthy^13^, Andy Rimmer^14^, Natasha Sahgal^1^, Ben Wright^1^, Chris Yau^7^

*^1^ The Wellcome Trust Centre for Human Genetics, Roosevelt Drive, Oxford, OX3 7BN, UK.*

*^2^ Office of the Regius Professor of Medicine, Richard Doll Building, Roosevelt Drive, Oxford,
OX3 7LF, UK*

*^3^ Illumina Cambridge Ltd., Chesterford Research Park, Little Chesterford, Essex, CB10 1XL, UK*

*^4^ NIHR Oxford Biomedical Research Centre, Oxford, UK.*

*^5^ Weatherall Inst of Molecular Medicine, University of Oxford; John Radcliffe Hospital*

*Headington, Oxford OX3 9DS, UK*

*^6^ Imperial College London, South Kensington Campus, London, SW7 2AZ. UK*

*^7^Centre for Computational Biology, Haworth Building, University of Birmingham, Edgbaston, Birmingham B15 2TT, UK*

*^8^UPMC Paris 6, CNRS UMR 7009, Villefranche-sur-Mer*

*^9^Oxford Nanopore Technology, Edmund Cartwright House, 4 Robert Robinson Avenue, Oxford Science Park, Oxford, OX4 4GA, UK*

*^10^CRUK Oxford Centre, Department of Oncology, Old Road Campus Research Building, Roosevelt Drive
Oxford, OX3 7DQ, UK*

*^11^Centre for Evolution and Cancer, The Institute of Cancer Research, Brookes Lawley Building, 15 Cotswold Road, Sutton, SM2 5NG, UK*

*^12^Wellcome Trust Sanger Institute, Wellcome Genome Campus, Hinxton, Cambridge, CB10 1SA, UK*

*^13^EMBL-EBI, Wellcome Genome Campus, Hinxton, Cambridgeshire, CB10 1SD, UK*

*^14^Genomics plc, King Charles House, Park End Street, Oxford, OX1 1JD, UK*

*^15^Imperial College London, Commonwealth Building, Hammersmith Campus, Du Cane Road, London, W12 0NN, UK*

*^16^Polanco-Echeverry G. Genome Center and Department of Biochemistry and Molecular Medicine, School of Medicine, University of California, Davis, USA*
